# Global FKRP Registry: observations in more than 300 patients with Limb Girdle Muscular Dystrophy R9

**DOI:** 10.1002/acn3.51042

**Published:** 2020-04-28

**Authors:** Lindsay B. Murphy, Olivia Schreiber‐Katz, Karen Rafferty, Agata Robertson, Ana Topf, Tracey A. Willis, Marcel Heidemann, Simone Thiele, Laurence Bindoff, Jean‐Pierre Laurent, Hanns Lochmüller, Katherine Mathews, Claudia Mitchell, John Herbert Stevenson, John Vissing, Lacey Woods, Maggie C. Walter, Volker Straub

**Affiliations:** ^1^ John Walton Muscular Dystrophy Research Centre Translational and Clinical Research Institute Newcastle University Newcastle upon Tyne UK; ^2^ Department of Neurology Hannover Medical School Hannover Germany; ^3^ Institute of Population Health Sciences University of Liverpool Liverpool UK; ^4^ The Robert Jones and Agnes Hunt Orthopaedic Hospital Oswestry UK; ^5^ Department of Neurology Friedrich‐Baur‐Institute Ludwig‐Maximilians‐University Munich Germany; ^6^ Department of Neurology Neuro‐SysMed Haukeland University Hospital University of Bergen Bergen Norway; ^7^ Department of Clinical Medicine University of Bergen Bergen Norway; ^8^ LGMD2i Research Fund Bellevue Washington; ^9^ Division of Neurology Department of Medicine Children's Hospital of Eastern Ontario Research Institute The Ottawa Hospital Ottawa Canada; ^10^ The Brain and Mind Research Institute University of Ottawa Ottawa Canada; ^11^ Department of Pediatrics University of Iowa Hospitals and Clinics Iowa City Iowa; ^12^ Astellas Pharma Tokyo Japan; ^13^ Cure LGMD2i University of Massachusetts Medical School Worcester Massachusetts; ^14^ Department of Neurology Copenhagen Neuromuscular Centre Rigshospitalet University of Copenhagen Copenhagen Denmark; ^15^ Patient Representative Stanwood Washington

## Abstract

**Objective:**

The Global FKRP Registry is a database for individuals with conditions caused by mutations in the *Fukutin‐Related Protein* (*FKRP*) gene: limb girdle muscular dystrophy R9 (LGMDR9, formerly LGMD2I) and congenital muscular dystrophies MDC1C, Muscle–Eye–Brain Disease and Walker–Warburg Syndrome. The registry seeks to further understand the natural history and prevalence of FKRP‐related conditions; aid the rapid identification of eligible patients for clinical studies; and provide a source of information to clinical and academic communities.

**Methods:**

Registration is patient‐initiated through a secure online portal. Data, reported by both patients and their clinicians, include: age of onset, presenting symptoms, family history, motor function and muscle strength, respiratory and cardiac function, medication, quality of life and pain.

**Results:**

Of 663 registered participants, 305 were genetically confirmed LGMDR9 patients from 23 countries. A majority of LGMDR9 patients carried the common mutation c.826C > A on one or both alleles; 67.9% were homozygous and 28.5% were compound heterozygous for this mutation. The mean ages of symptom onset and disease diagnosis were higher in individuals homozygous for c.826C > A compared with individuals heterozygous for c.826C > A. This divergence was replicated in ages of loss of running ability, wheelchair‐dependence and ventilation assistance; consistent with the milder phenotype associated with individuals homozygous for c.826C > A. In LGMDR9 patients, 75.1% were currently ambulant and 24.6%, nonambulant (unreported in 0.3%). Cardiac impairment was reported in 23.2% (30/129).

**Interpretation:**

The Global FKRP Registry enables the collection of patient natural history data, which informs academics, healthcare professionals and industry. It represents a trial‐ready cohort of individuals and is centrally placed to facilitate recruitment to clinical studies.

## Introduction

In recent years, advances in potential treatments for rare neuromuscular disorders have gained pace with the development of multiple molecular therapies. In conjunction with these developments, the relevance and importance of patient registries as a tool to assist patient location and recruitment have become firmly established.

The Global FKRP Registry collects clinical and genetic data from individuals with mutations in the *Fukutin‐Related Protein* (*FKRP*) gene. *FKRP* mutations cause a number of rare, autosomal recessive muscular dystrophies (MDs), the most common of which is limb girdle muscular dystrophy R9, FKRP‐related (LGMDR9), formerly named LGMD2I and muscular dystrophy‐dystroglycanopathy type C, 5 (MDDGC5) (OMIM#607155). *FKRP* mutations are also the cause of the rarer congenital conditions congenital muscular dystrophy type 1C (MDC1C), muscle–eye–brain disease (MEB), and Walker–Warburg syndrome (WWS), which present with a more severe phenotype.^1^ FKRP‐related MDs are highly variable in presentation, as might be expected when comparing the congenital conditions to the later onset LGMDR9. However, even within LGMDR9, there is considerable variation in disease onset and progression, ranging from a childhood‐onset, Duchenne‐like progression, to mild, adult onset phenotypes with preserved walking ability for decades.^2^, ^3^ There is currently no curative treatment for FKRP‐related MDs, with many affected individuals losing mobility, requiring ventilation and cardiac intervention. Steroid treatment may be an option but until now only case reports have been published.^4^, ^5^, ^6^ Recent research indicates the efficacy of gene replacement therapy in a murine model,^7^, ^8^, ^9^, ^10^ leading to the introduction of gene therapy development programmes in LGMDR9. Encouraging preclinical research in gene replacement therapy and pluripotent stem cell therapy^11^ demonstrate the increasing need to prepare for potential clinical trials and ensure that eligible patient cohorts can be identified for these novel therapies.

Drug development for rare diseases is associated with many and specific challenges, including low patient numbers available for study, limited knowledge of the disease’s natural progression and heterogeneity of the disease. Patient registries help to surmount these challenges by providing a useful tool to support and facilitate drug development and research. In 2011, the Global FKRP Registry was established to resolve the challenges of data fragmentation and lack of infrastructure for trial‐readiness in FKRP‐related MDs. The rarity of FKRP‐related MDs favored the establishment of an international registry rather than national registries. The registry provides a source of information to assist research, for example, in the identification of appropriate outcome measures and the understanding of underlying pathologies; to support healthcare professionals in the development of standards of care; to disseminate relevant FKRP‐related information and support the FKRP community. The Global FKRP Registry was created under the auspices of the TREAT‐NMD Alliance.^12^, ^13^ TREAT‐NMD has long championed patient registries, exemplifying their use in facilitating planning and recruitment for clinical trials for rare inherited neuromuscular diseases (for example, Duchenne muscular dystrophy (DMD) and spinal muscular atrophy (SMA)), their successful utilization by both researchers and industry, and their assistance in gaining a better understanding of the natural history of rare diseases.^14^, ^15^, ^16^, ^17^ As novel therapies progress to trial and ultimately to market, registries are entering a new phase in their evolution by their utilization to capture pharmacovigilance and long‐term efficacy data.

This article describes the Global FKRP Registry and the characteristics of its patient cohort, obtained from the registry’s inception in April 2011–March 2019. In March 2019, the overall Global FKRP Registry population was 663 participants. Here we provide cross‐sectional analysis on patients with genetically confirmed FKRP‐related MD (*n* = 320/663) and, more particularly, on the genetically confirmed LGMDR9 cohort (305).

## Subjects and Methods

**Figure 1:**
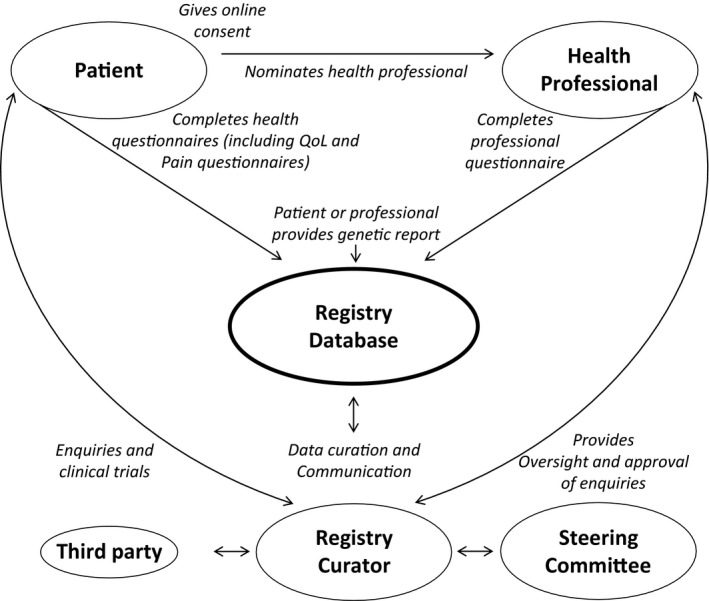
Registration process and data flow in the Global FKRP Registry.

The Global FKRP Registry is an international, online registry, accessed at http://www.fkrp-registry.org, which captures clinical and genetic information entered by both patients and their nominated clinicians via the same interface (Fig. 1). Patients initiate registration and provide consent online. They are then asked to complete a short mandatory questionnaire about the current status of their health and two optional validated questionnaires on quality of life (INQoL^18^) and pain (McGill^19^) (copyright for questionnaire use was obtained). The mandatory dataset for patients was developed with input from key opinion leaders, genetic centers, patients, and patient organization representatives, and focuses on the key items which help to carry out feasibility studies and recruitment for clinical trials (Table 1).

**Table 1:** The clinical dataset captured in the Global FKRP Registry.

Patient‐reported data	Professional‐reported data
Demographics^c^	Disease onset
Diagnosis^b^	Age^c^
Motor function	Symptoms^b^
Current best motor function^a,^*	Respiratory function*
Previous best motor function^a^	FVC^b^
Wheelchair use^a,^*	Cardiac function*
Myalgi^a,^*	Echo^b^
Ventilation*	MRI*
Noninvasive^a^	Brain^b^
Invasive^a^	Muscle^b^
Family history^a^	Cognitive function^a^
Quality of life (INQoL)^a,^*	Contractures^b,^*
McGill Pain Questionnaire (SF‐MPQ)^a,^*	Other medical problems^b^
	Current medication^b^
	Muscle strength and function*
	6MWD^c^
	MRC scores^c^
	Genetic mutation^b^

Data are defined as being either patient‐reported or professional‐reported and data items collected longitudinally are labeled with *. The reporting options available for each data item are indicated: ^a^drop‐down menu only; ^b^drop‐down menu with option to add additional information as free text; and ^c^free text only. Among all registry participants, completion of patient‐reported data items ranges between 87 and 95%. Completion rates of INQoL and SF‐MPQ are approximately 75%. A doctor has been selected by 40% (267/663) of all registry participants, 42% (111/267) of whom have entered data on behalf of their patient. Participation is increased among patients with genetic confirmation of *FKRP* mutation: completion of patient‐reported data items ranges between 98 and 100%. Completion rates of INQoL and SF‐MPQ are approximately 95%. A doctor has been selected by 56% (180/320) of genetically confirmed participants, 58% (105/180) of whom have entered data on behalf of their patient. Medical patient records are the source of professional‐reported data. When considering family history, patients are asked only whether a family member has been diagnosed with an FKRP‐related condition. Therefore, with the exception of child siblings registered by the same parent/guardian, family links between registry participants are not readily available.

It is a special feature of the Global FKRP Registry that during the registration process, patients indicate their respective neuromuscular specialist from a prepopulated list and provide consent for that clinician to enter their medical data into the registry. The nominated clinicians can view and add clinical and genetic information for the patients that have selected them. A yearly information update is requested from patients and clinicians for longitudinal data capture. This allows for collection of data over time to provide a more detailed clinical picture of disease progression with regards to genotype.

The registry uses bespoke software designed at the Friedrich‐Baur‐Institute of Ludwig‐Maximilians‐University Munich. The Java EE‐based web application covers both the patient identity and medical data management by curators as well as a user‐friendly interface for patients and clinicians. The registry holds favorable ethical opinions from the “Ethikkommission der Medizinischen Fakultät der LMU München”, for Germany and from the NRES Committee North East – Newcastle and Tyneside 1, for the United Kingdom (UK). A steering committee, including neuromuscular specialists, geneticists and patient representatives, oversees governance of the registry and ensures that all enquiries are in line with its purpose and in the best interests of the patients. All requests into the registry from third parties are considered and must be approved by the steering committee. Only anonymous and aggregate information on patients in the registry is shared with third parties. Recruitment into clinical trials is implemented by the registry curator who identifies and contacts potentially eligible patients, effectively ensuring that pharmaceutical companies never receive patient contact details directly. Statistical analysis was performed using SPSS^®^ software.

## Results

### Patient demographics

Six hundred and sixty‐three patients with FKRP‐related MDs worldwide registered with the Global FKRP Registry between April 2011 and March 2019. The largest cohort of patients reported a diagnosis of LGMDR9 (586, 88.4%). A further 2.0% (13) reported a diagnosis of MDC1C and 4.1% (27) of an unspecified FKRP‐related MD. A diagnosis was not reported in 5.5% (37) registrations. Genetic confirmation was pending for a proportion of patients due to a number of factors including restricted availability of genetic testing in some countries. In its absence, it was not possible for the registry to confirm that a patient has an FKRP‐related condition. Therefore, only data from patients with confirmed *FKRP* mutation are here presented.

**Figure 2:**
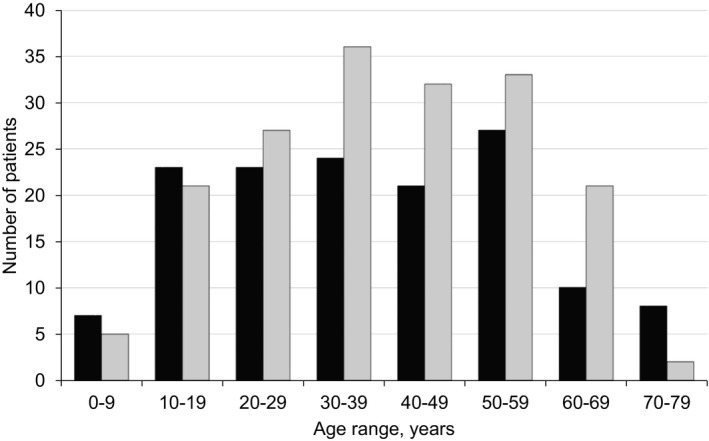
The current age of all genetically confirmed Global FKRP Registry participants (*n* = 320). The number of male and female patients within each 10‐year age range, from 1 to 9 through to 70–79 years, is presented. Male (black); female (gray).

Three hundred and twenty registry participants had genetic confirmation of their FKRP‐related MD, of which 305 (95.3%; 168 female, 137 male) were diagnosed with LGMDR9 and 15 (4.9%; 9 female, 6 male), with another form of FKRP‐related MD. Genetically confirmed patients originated from 23 countries, the majority of whom were from Germany (96; 30.0%); United States of America (USA, 69; 21.6%); UK (50; 15.6%); and Denmark (31; 9.7%). The age range of genetically confirmed patients was normally distributed and the largest number of patients were between 10 and 59 years of age (Fig. 2). Our descriptive analyses focus on the 305 patients with genetically verified diagnosis of LGMDR9.

### Genetic mutations

**Figure 3:**
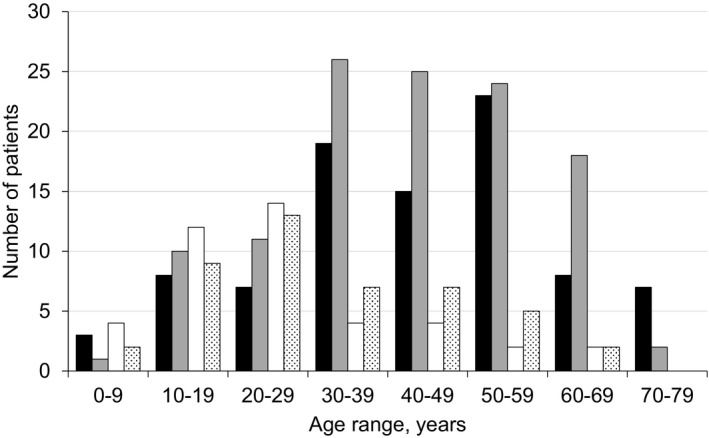
The current age of LGMDR9 patients with the common *FKRP* gene mutation (c.826C > A, *n* = 294). Patient number within each 10‐year age range is further stratified by zygosity of the common *FKRP* gene mutation and by sex. The difference between homozygous c.826C > A and heterozygous c.826C > A mean ages is significant (*P* < 0.0001). Homozygous c.826C > A, male (black); Homozygous c.826C > A, female (gray); Heterozygous c.826C > A, male (white); Heterozygous c.826C > A, female (dots).

A large variety of genetic mutations in the *FKRP* gene was reported in the registry. A majority of LGMDR9 patients was homozygous for the common mutation (c.826C > A; 207, 67.9%), followed by compound heterozygosity for the common mutation (87, 28.5%) and compound heterozygosity for mutations other than c.826C > A (8, 2.6%). Three patients were homozygous for a mutation other than c.826C > A (1.0%) (Table S1). Patients who did not possess the c.826C > A mutation were from Australia (1), Canada (4), France (1), Germany (1), Spain (1), Sweden (1), and the USA (2). Among the compound heterozygous mutations, the most frequently found on the second allele were c.919T > A (4), c.1384C > T (4), c.1073C > T (4), and c.586G > C (4). The age distribution of LGMDR9 patients with at least one allele of the common mutation is described (Fig. 3). Patients homozygous for the common allele had a relatively normal age distribution with greatest numbers between the ages of 30 and 59 years. In contrast, in individuals heterozygous for the common mutation, the distribution curve was shifted to the left, with greatest numbers occurring between the ages of 10 and 29 years, and group mean ages were significantly different (*P* < 0.0001), indicative of the patient cohort being affected by greater disease severity. There are 30 novel mutations associated with a disease phenotype reported in the registry, which have not previously been reported in the Leiden Open‐source Variation Database (LOVD) or in ClinVar.

### Disease onset, diagnosis and presenting symptoms in LGMDR9

In LGMDR9 patients, the mean age when symptoms first presented, or were incidentally found, was 15.1 ± 12.3 years (152 patients). However, patients with the homozygous common mutation (102) appeared to present at the mean age of 19.0 ± 12.4 years, a range of 7.0–13.2 years later than patients who carried at least one disease allele different from the common mutation (*P* < 0.0001) (Table S2). Age of disease onset was reported by clinicians and was therefore not available for all patient records analyzed. The most frequently reported presenting symptoms were weakness in lower limbs (107), proximal weakness (69) and hyperCKaemia (52). The mean age of disease onset for each of these symptoms was 17.2 ± 12.8, 18.1 ± 12.6 and 11.0 ± 8.8 years, respectively. LGMDR9 individuals who were homozygous for the common mutation presented later than average for these symptoms, consistent with their presentation with a milder phenotype. Of the patients presenting with hyperCKaemia, 29 patients had the homozygous common mutation, 19 were heterozygous for the common mutation, two patients were heterozygous, and two were homozygous for a mutation other than c.826C > A. In this snapshot analysis, the mean age of diagnosis for all LGMDR9 patients (defined as the age when a genetic confirmation was received) was 30.1 ± 17.3 years, significantly earlier than for LGMDR9 patients with the homozygous common mutation whose mean age of diagnosis was 34.8 ± 15.9 years (*P* = 0.005). This followed the trend for later disease onset in this patient cohort.

### Motor function

The registry collected data on both the previous best motor function and the current motor function of patients. The classification of motor function was divided into ambulant (run, climb stairs with/out assistance, walk with/out assistance) and nonambulant (sit independently, neither able to sit nor walk independently). Among genetically confirmed LGMDR9 patients, 303 (99.3%) reported having been ambulant at some stage in their life. Two hundred and twenty‐nine (75.1%) LGMDR9 patients were currently ambulant and 75 (24.6%) were nonambulant (unreported by one individual (0.3%)). Though captured by the registry and reported by the majority of patients, longitudinal data were not considered in this analysis and snapshot analyses of current data only are here presented.

#### Previous best motor function

**Figure 4:**
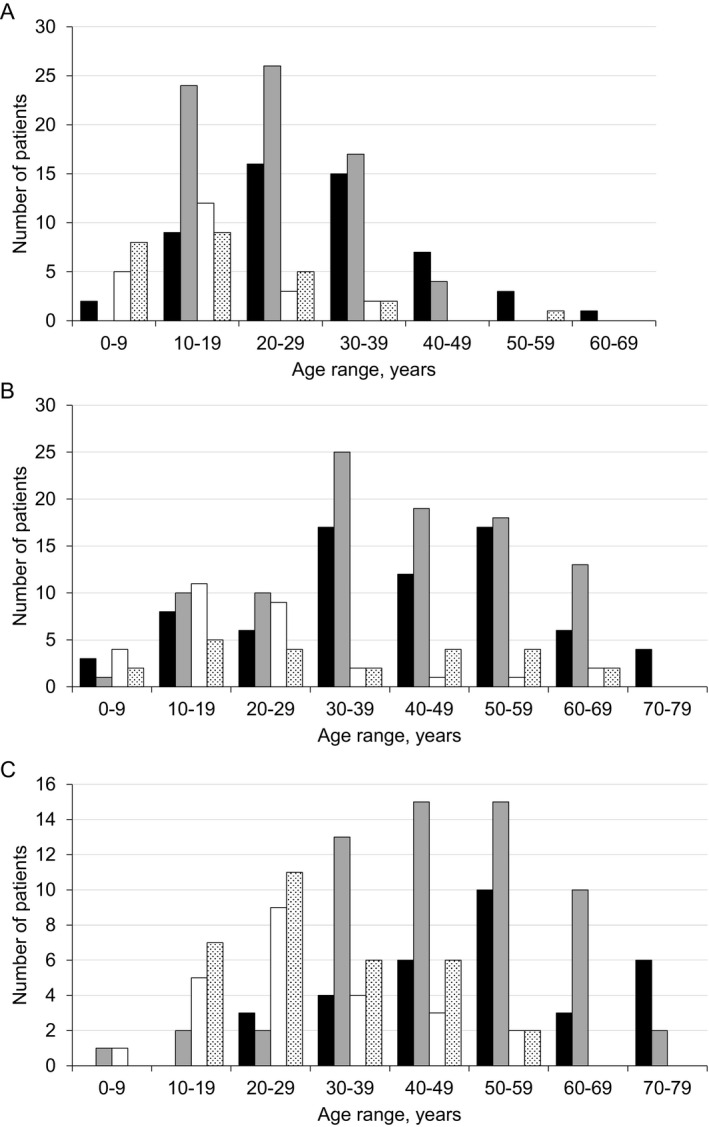
The age of LGMDR9 patients with the common *FKRP* gene mutation associated with specific motor functions: (A) the age of LGMDR9 patients at which running ability was lost (*n* = *171*). The difference between homozygous c.826C > A and heterozygous c.826C > A mean ages is significant (*P* < 0.0001); (B) the current age of ambulant LGMDR9 patients (*n* = 222); and (C) the current age of LGMDR9 patients who use a wheelchair, either part‐time or full‐time (*n* = 148). For each graph, patient number within each 10‐year age range is further stratified by zygosity of the common *FKRP* gene mutation and by sex. Homozygous c.826C > A, male (black); Homozygous c.826C > A, female (gray); Heterozygous c.826C > A, male (white); Heterozygous c.826C > A, female (dots).

Analysis of the previous best motor function indicated that the majority of genetically confirmed LGMDR9 patients (234/305, 76.7%) were able to run in the past. This ability was achieved in an equal proportion of patients homozygous and compound heterozygous for the common mutation (80.6%, 166/206 vs. 70.1%, 61/87, *P* = 0.059). In both genotype groups, the ability to run diminished and was lost in the first few decades of life with the majority having lost it by the age of 40–49 years (Fig. 4A). Within the group, loss of running ability by individuals compound heterozygous for the common mutation occurred earlier than in individuals homozygous for the common mutation (average age of 15.9 ± 10.2 years compared with 26.2 ± 10.7 years, respectively, *P* < 0.0001). By the age of 29 years, 91.8% of the group compound heterozygous for the common mutation had lost their ability to run. In homozygous females, running ability was lost earlier than in their male counterparts, with 70.4% of females losing this ability before the age of 29 years compared with 50.9% of males (*P* = 0.027). Phenotypic differences between the sexes have previously been reported^20^ though not a difference in disease severity. Within patient‐reported data, elements of bias in patient selection, reporting and recall may occur that impact on the collective dataset. Though its potential influence may be difficult to identify, it should be acknowledged.

#### Current motor function – ambulation in LGMDR9

Analysis of the current motor function showed a general trend of older patients being less mobile, mirroring the trend within the wider, healthy population.

Cross‐sectional analysis of genetically confirmed LGMDR9 registry participants indicated that 42/304 (13.8%) were currently able to run: 28 (9.2%) with the homozygous common mutation (age < 39 years); 13 (4.3%) with the heterozygous common mutation (age < 25 years); and one (0.3%) with a heterozygous unique mutation (age = 12 years). This further suggests that patients with compound heterozygous mutations showed a more rapid disease progression, expressed as mobility challenges earlier in life.

One hundred and eighty‐seven (61.5%) LGMDR9 patients reported that they were currently able to climb stairs or walk with or without support, the oldest of whom was 76 years old. This cohort included 141 (46.4%) patients with the homozygous common mutation, 40 (13.1%) with one common mutation, three patients (1.0%) with unique homozygous, and three (1.0%) with unique heterozygous mutations. The mean age of patients with homozygous common mutation with preserved ability to climb stairs (59/141) was 43.5 ± 14.6 years. As anticipated, this was lower than the mean age of patients of this mutation type whose current best motor function was walking (82/141; 47.0 ± 13.4 years). The mean age of patients with one common mutation who were able to climb stairs (15/40) was 25.5 ± 16.9 years compared with that of those able to walk (25/40), which was 37.2 ± 18.7 years. These data indicate that individuals with a single common mutation lose their ability to climb stairs (*P* < 0.0001) and walk (*P* = 0.005) at an earlier age than those with two common mutations. The current ambulatory status of LGMDR9 patients with the common mutation is summarized in Figure 4B.

#### Current motor function – loss of ambulation in LGMDR9

Among LGMDR9 patients with the homozygous common mutation, 92/207 (44.4%) were wheelchair‐dependent (full‐time 38/92, mean age 53.2 ± 12.7 years; part‐time 54/92, mean age 45.8 ± 15.8 years). Among patients who were heterozygous for the common mutation, 56/87 (64.4%) were wheelchair‐dependent (full‐time 35/56, mean age 32.6 ± 12.0 years; part‐time 21/56, mean age 25.4 ± 13.6 years) (Fig. 4c). Comparison between each patient group shows significant differences in mean age of part‐time wheelchair users (*P* < 0.0001) and full‐time wheelchair users (*P* < 0.0001).Once again, this underlines the faster disease progression in heterozygous patients who became wheelchair‐dependent earlier in life in comparison with homozygous patients.

### Ventilation

Breathing complications are common in FKRP‐related MDs. Therefore, the registry collected data on the respiratory impairment of patients and their use of assisted ventilation.

#### Noninvasive ventilation

Within the genetically confirmed LGMDR9 patient cohort, 46 patients (15.1%) used noninvasive ventilation, 39 (12.9%) of whom reported the age at which they began respiratory support. The average age of starting noninvasive ventilation was 40.8 ± 15.0 years, with differences between the genotypes. In the group compound heterozygous for the common mutation, respiratory function appeared to be impaired earlier in life, with a mean age of starting noninvasive ventilation of 32.7 ± 13.2 years (12 patients) compared with the patients homozygous for the common mutation whose equivalent mean age was 46.6 ± 13.7 years (24 patients, *P* = 0.006) (Table S2).

#### Invasive ventilation

Nine patients (2.9%) reported receipt of invasive ventilation with the mean age of commencement being 43.1 ± 10.3 years (age reported by six patients). The heterozygous common mutation patient population started invasive ventilation at an earlier mean age of 39.3 ± 5.0 years (5 patients). In comparison, only one patient homozygous for the common mutation started invasive ventilation, aged 62 years.

### Cardiac function

Within the registry, information about patients’ cardiac function was provided by clinicians. There was information about cardiac function for 129/305 genetically confirmed LGMDR9 patients in the registry, of whom 82 (63.6%) showed normal results at their last cardiac assessment and 30 (23.2%) showed some cardiac impairment. Cardiac function was not specified for 17 patients (13.2%) as these records were unavailable to the reporting clinician. Further breakdown indicated that, of 30 individuals with impaired cardiac function, 18 had started treatment, two showed cardiac deterioration and had their medication changed as a result, and 10 were not on treatment. Of the 129 LGMDR9 patients for whom cardiac information was available, 29 (22.5%) individuals took ACE inhibitors and 16 (12.4%) were prescribed beta blockers.

## Discussion

The Global FKRP Registry captures a wealth of data, including patients’ current disease status and their longitudinal history. The registry holds data reported by both patients and clinicians, which enhances its validity and helps reduce the requirement for lengthy curation time, often required by registries that capture patient‐entered data only. This combined reporting method enables patients to drive the process and register themselves while also ensuring the capture of high quality data. Patients are able to act as a catalyst to prompt their clinician if data are not entered.

The Global FKRP Registry is currently available in English, German, Norwegian, and Portuguese. This limited availability of languages may present a barrier to registration for some patients. Furthermore, the registry’s name, the Global FKRP Registry, may not be immediately recognizable to patients, as it includes the name of the causative gene rather than those of the associated diseases. Unless directed by their clinician, many patients will not initially know that their condition is caused by a mutation in the *FKRP* gene. Patient awareness is therefore still a substantial challenge for the registry in terms of patient recruitment. This is addressed by presenting the registry at various scientific and patient conferences, by patient organizations emphasizing the value of the registry in LGMDR9‐specific social media groups and through direct communication.

Worldwide, clinicians have limited time available to enter patient data, therefore, obtaining complete datasets is challenging. To facilitate this process, the steering committee has reviewed and amended the mandatory questionnaires for both patients and clinicians to maximize the data that can be entered by patients and capitalize on their motivation. As genetic information is reported by clinicians, it is difficult to ensure that all patients do indeed have an FKRP‐related MD at the time of registration. Encouraging patients to send their own genetic report directly to the registry both reduces this burden from clinicians and curation time.

The analysis of clinical and genetic data from the Global FKRP Registry has demonstrated that LGMDR9 patients who are homozygous for the common mutation (c.826C > A) are more likely to have a milder phenotype and have a later disease onset than those patients who are compound heterozygous for the common mutation, or have a mutation other than the common mutation. Grouping of the data by the presence or absence of the common mutation may be a generalization but reflects broad phenotypic observations of the LGMDR9 cohort and paves the way for more detailed investigation of the association between *FKRP* gene mutation and disease phenotype. It is important to bear in mind that the congenital FKRP‐related MDs are underrepresented in the registry due to their severity and early onset. This contributed to the selection of LGMDR9 alone as the focus of this article.

Analysis of the registry data indicates that disease symptoms presented on average between 7.0 and 13.2 years later in patients with LGMDR9 who were homozygous for the common mutation; with weakness in lower limbs, proximal weakness and hyperCKaemia being the most common presenting symptoms and affecting more than 30% of all genetically confirmed LGMDR9 patients. The average age of disease diagnosis was 30.1 ± 17.3 years. This varied depending on the causative genetic mutation, with patients who were homozygous for the common mutation being diagnosed around 4.5 years later (34.8 ± 15.9 years).

The walking ability of patients was variable across the range of ages, with no specific, well‐defined period at which there was a greater probability of becoming nonambulant. LGMDR9 patients tended to retain the ability to walk into their fifties, even into their seventies. However, in this analysis of current data only, it was not possible to determine the exact age at which loss of ambulation occurred. Past best and current motor functions and the age at which they were gained/lost are reported by patients on an annual basis. Therefore, data enabling the rate of disease progression to be studied are potentially captured, however, they are not included in this analysis. In addition, greater granularity in questionnaire answers would allow a more accurate prediction of the course of disease progression.

Fifty‐five genetically confirmed LGMDR9 patients (55/303, 18.2%) included in this snapshot analysis required ventilation, both noninvasive (46/303; 15.2%) and invasive (9/303; 3.0%), and 30/129 (23.3%) reported a heart condition. This demonstrates the importance of monitoring both pulmonary and cardiac function as routine aspects of care for the FKRP‐related MDs. It is recognized that LGMDR9 may sometimes follow the presentation of DMD. Therefore, the care standards for DMD^21^, ^22^ should be followed, which include monitoring heart and lung function to enable intervention before significant deterioration occurs, as is the same with the congenital muscular dystrophies.^23^, ^24^ Further analysis with greater numbers of patients may allow predictions of which subpopulations are at greater risk of developing heart and/or lung complications, based on their specific genetic mutations.

Recent advances in neuromuscular disease‐specific therapies, spearheaded by novel gene therapy developments in DMD and SMA, reflect the current excitement and optimism within the field. For LGMDR9, the Global FKRP Registry has facilitated recruitment to a number of natural history and research studies and clinical trials. However, the development of new therapies introduces the new challenge of capturing postmarketing surveillance data, a task the registry is well placed to accommodate. It is intended that presentation of this snapshot analysis of LGMDR9 will add to the growing body of knowledge of the condition and support the establishment of bespoke LGMD standards of care.

## Conflict of Interest

The authors report no conflicts of interest relating to this work.

## Author contributions

Volker Straub and Maggie Walter supervise the Global FKRP Registry research project. In collaboration with Hanns Lochmuller, they were responsible for its conception and design. Lindsay Murphy and Olivia Schreiber‐Katz contributed equally to the analysis and interpretation of the data and the drafting of the manuscript. Ana Topf also contributed to data analysis and interpretation. Karen Rafferty, Agata Robertson and Tracey Willis assisted the drafting of the manuscript. Marcel Heidemann and Simone Thiele facilitated the access to data. The registry steering committee, past and present, provided critical review of the manuscript for important intellectual content: Laurence Bindoff, Jean‐Pierre Laurent, Katherine Mathews, Claudia Mitchell, J. Herbert Stevenson, John Vissing, and Lacey Woods.

## Supporting information


**Table S1**. All *FKRP* gene mutations associated with LGMDR9 reported in the Global FKRP Registry (*n* = *305*).

**Table S2**. Clinical data of all patients in the Global FKRP Registry. Included are data of registry patients with genetic confirmation (*n* = *320*) and without genetic confirmation (*n* = *343*).
