# Supplementary material for: Global FKRP Registry: observations in more than 300 patients with Limb Girdle Muscular Dystrophy R9
Source: Ann Clin Transl Neurol. 2020 Apr 28;7(5):757–66. doi: 10.1002/acn3.51042 (PMC7261761; doi:10.1002/acn3.51042)
Supplement: Supplementary file 1 — Table S1. All FKRP gene mutations associated with LGMDR9 reported in the Global FKRP Registry (n = 305). [file ACN3-7-757-s001.pdf]

No. of patients	Nucleotide change - allele 1	Effect on FKRP protein sequence 1	Novel mutation - allele 1	Nucleotide change - allele 2	Effect on FKRP protein sequence 2	Novel mutation - allele 2
206	c.826C>A	p.Leu276Ile	No	c.826C>A	p.Leu276Ile	No
1	c.826C>A	p.Leu276Ile	No	c.826C>A<br>c.390insTACC	p.Leu276Ile<br>p.Asp131TyrfsTer7	No<br>Yes
4	c.826C>A	p.Leu276Ile	No	c.586G>C	p.Gly196Arg	No
4	c.826C>A	p.Leu276Ile	No	c.1384C>T	p.Pro462Ser	No
4	c.826C>A	p.Leu276Ile	No	c.1073C>T	p.Pro358Leu	No
4	c.826C>A	p.Leu276Ile	No	c.919T>A	p.Tyr307Asn	No
3	c.826C>A	p.Leu276Ile	No	c.229C>T	p.Gln77*	Yes
2	c.826C>A	p.Leu276Ile	No	c.1187insA	p.Ala397Glyfs*67	No
2	c.826C>A	p.Leu276Ile	No	c.1486T>A	p.*496Argext*21	No
2	c.826C>A	p.Leu276Ile	No	c.962C>A	p.Ala321Glu	No
2	c.826C>A	p.Leu276Ile	No	c.1016G>A	p.Arg339His	No
2	c.826C>A	p.Leu276Ile	No	c.1088T>G	p.Val363Gly	Yes
2	c.826C>A	p.Leu276Ile	No	c.946C>T	p.Pro316Ser	No
2	c.826C>A	p.Leu276Ile	No	c.928G>T	p.Glu310*	No
2	c.826C>A	p.Leu276Ile	No	c.469G>C	p.Ala157Pro	No
2	c.826C>A	p.Leu276Ile	No	c.532T>G	p.Trp178Gly	Yes
2	c.826C>A	p.Leu276Ile	No	c.646C>T	p.Arg216Trp	Yes
2	c.826C>A	p.Leu276Ile	No	c.545A>G	p.Tyr182Cys	No
2	c.826C>A	p.Leu276Ile	No	c.217C>T	p.Gln73*	Yes
1	c.826C>A	p.Leu276Ile	No	c.1217A>C	p.Gln406Pro	Yes
2	c.826C>A	p.Leu276Ile	No	c.1054C>G	p.Arg352Gly	No
1	c.826C>A	p.Leu276Ile	No	c.1054C>T	p.Arg352Cys	No
1	c.826C>A	p.Leu276Ile	No	c.1054C>A	p.Arg352Ser	Yes
1	c.826C>A	p.Leu276Ile	No	c.673C>T	p.Gln225*	Yes
1	c.826C>A	p.Leu276Ile	No	Not specified	Not specified	-
1	c.826C>A	p.Leu276Ile	No	c.1384C>T<br>c.341C>G	p.Pro462Ser<br>p.Ala114Gly	No<br>No
1	c.826C>A	p.Leu276Ile	No	c.341C>G	p.Ala114Gly	No
1	c.826C>A	p.Leu276Ile	No	c.135C>T<br>c.341C>G<br>c.1486T>A	p.Ala45Ala<br>p.Ala114Gly<br>p.Stop496Arg	No<br>No<br>No
1	c.826C>A	p.Leu276Ile	No	c.426_437del	p.Arg143_Glu146del	No
1	c.826C>A	p.Leu276Ile	No	c.1037C>T	p.Ser346Leu	Yes
1	c.826C>A	p.Leu276Ile	No	c.1381G>C	p.Ala461Pro	Yes
1	c.826C>A	p.Leu276Ile	No	c.948_949dupC<br>c.1000_1017dup18	p.Cys317Serfs*112<br>p.Glu334_Arg339dup	Yes<br>No
1	c.826C>A	p.Leu276Ile	No	c.934C>G	p.Arg312Gly	Yes
1	c.826C>A	p.Leu276Ile	No	c.430A>G	p.Met144Val	No
1	c.826C>A	p.Leu276Ile	No	c.362T>A	p.Val121Glu	No
1	c.826C>A	p.Leu276Ile	No	c.398C>A	p.Ala133Glu	Yes
1	c.826C>A	p.Leu276Ile	No	c.1268G>C	p.Arg423Pro	No
1	c.826C>A	p.Leu276Ile	No	c.391G>A	p.Asp131Asn	Yes
1	c.826C>A	p.Leu276Ile	No	c.88C>T	p.Gln30*	Yes
1	c.826C>A	p.Leu276Ile	No	c.214C>T	p.Gln72*	No
1	c.826C>A	p.Leu276Ile	No	c.534G>T	p.Trp178Cys	Yes
1	c.826C>A	p.Leu276Ile	No	c.1000G>T	p.Glu334*	Yes
1	c.826C>A	p.Leu276Ile	No	c.1433T>C	p.Ile478Thr	No
1	c.826C>A	p.Leu276Ile	No	c.605T>A	p.Leu202Gln	No
1	c.826C>A	p.Leu276Ile	No	c.620T>C	p.Leu207Pro	Yes
1	c.826C>A	p.Leu276Ile	No	c.872delA	p.Lys291Argfs*137	No
1	c.826C>A	p.Leu276Ile	No	c.943C>T	p.Pro315Leu	Yes
1	c.826C>A	p.Leu276Ile	No	c.946C>G<br>c.970G>C	p.Pro316Ala<br>p.Glu324Gln	Yes<br>Yes
1	c.826C>A	p.Leu276Ile	No	c.836G>A	p.Trp279*	No
1	c.826C>A	p.Leu276Ile	No	c.1076G>C	p.Trp359Ser	Yes
1	c.826C>A	p.Leu276Ile	No	c.899T>C	p.Val300Ala	No
1	c.826C>A	p.Leu276Ile	No	c.1253G>A	p.Trp418*	No
1	c.826C>A	p.Leu276Ile	No	c.797insC	p.Ala267Glyfs*123	Yes
1	c.826C>A	p.Leu276Ile	No	c.158_162dupTGCGG	p.Glu55Cysfs*15	No
1	c.826C>A	p.Leu276Ile	No	c.162_165dupGGAG	p.Phe56Glyfs*6	No
1	c.826C>A	p.Leu276Ile	No	c.1171G>A	p.Gly391Ser	No
1	c.826C>A	p.Leu276Ile	No	c.76_77delTG	p.Trp26Alafs*6	No
1	c.826C>A	p.Leu276Ile	No	c.1115T>G	p.Val372Gly	No
1	c.826C>A	p.Leu276Ile	No	c.1141delG	p.Ala381Glnfs*47	No
1	c.826C>A	p.Leu276Ile	No	c.264C>G	p.Tyr88*	No
1	c.826C>A	p.Leu276Ile	No	c.160C>G	p.Arg54Gly	Yes
1	c.826C>A	p.Leu276Ile	No	c.266C>T	p.Pro89Leu	No
2	c.1100T>C	p.Ile367Thr	No	c.1100T>C	p.Ile367Thr	No
2	c.1388A>G	p.Asn463Ser	Yes	c.162_165dupGGAG	p.Phe56Glyfs*6	No
1	c.1486T>A	p.*496Argext*21	No	c.1486T>A	p.*496Argext*21	No
1	c.1073C>T	p.Pro358Leu	No	c.1210C>T	p.Arg404Cys	No
1	c.265C>T	p.Pro89Ser	Yes	c.1433T>G	p.Ile478Ser	No
1	c.266C>T	p.Pro89Leu	No	c.1247A>G	p.Asp416Gly	Yes
1	c.430A>G	p.Met144Val	No	c.469G>C	p.Ala157Pro	No
1	c.520A>T	p.Ser174Cys	No	Not specified	Not specified	-
1	c.1343C>T	p.Pro448Leu	Yes	c.1387A>G	p.Gln460Glu	Yes
