# Supplementary material for: Global FKRP Registry: observations in more than 300 patients with Limb Girdle Muscular Dystrophy R9
Source: Ann Clin Transl Neurol. 2020 Apr 28;7(5):757–66. doi: 10.1002/acn3.51042 (PMC7261761; doi:10.1002/acn3.51042)
Supplement: Supplementary file 2 — Table S2. Clinical data of all patients in the Global FKRP Registry. Included are data of registry patients with genetic confirmation (n = 320) and without genetic confirmation (n = 343). [file ACN3-7-757-s002.pdf]

Clinical characteristics and demographics	Patient Groups						
	Non-genetically confirmed patients	All genetically confirmed patients	All genetically confirmed LGMDR9 patients	Homozygous common in LGMDR9	Heterozygous common in LGMDR9	Homozygous unique in LGMDR9	Heterozygous unique in LGMDR9
**Patients (n)**<br>**Sex (f/m, n)**	343<br>183/160	320<br>177/143	-<br>-	-<br>-	-<br>-	-<br>-	-<br>-
**LGMD R9 patients (n)**<br>**Sex (f/m, n)**	-<br>-	-<br>-	305<br>168/137	207<br>117/90	87<br>45/42	3<br>1/2	8<br>5/3
Diagnosis							
**Mean age of genetically confirmed diagnosis (years ±SD, n)**<br>***(at time of diagnosis)***	-	29.9 ± 17.5 (259)<br>F(141): 30.8 ± 16.5<br>M(118): 29.0 ± 18.6	30.1 ± 17.3 (251)<br>F(138): 31.1 ± 16.4<br>M(113): 28.8 ± 18.3	34.8 ± 15.9 (164)<br>F(90): 35.1 ± 15.2<br>M(74): 34.5 ± 16.8	20.7 ± 16.5 (76)<br>F(42): 23.2 ± 16.2<br>M(34): 17.5 ± 16.5	19.2 ± 7.4 (3)<br>F(1): 17.6<br>M(2): 20.0 ± 10.3	26.1 ± 16.6 (8)<br>F(5): 29.3 ± 17.4<br>M(3): 20.7 ± 17.0
Disease onset							
**Mean age of symptom onset (years ±SD, n)**	9.0 ± 6.4 (4)<br>F(1): 7.0<br>M(3): 9.7 ± 7.6	14.8 ± 12.3 (155)<br>F(88): 14.2 ± 11.0<br>M(67): 15.7 ± 13.9	15.1 ± 12.3 (152)<br>F(86): 14.5 ± 10.9<br>M(66): 15.9 ± 13.9	19.0 ± 12.4 (102)<br>F(61): 17.6 ± 10.6<br>M(41): 21.0 ± 14.5	6.8 ± 7.3 (44)<br>F(22): 6.4 ± 7.2<br>M(22): 7.2 ± 7.5	5.8 ± 0.4 (2)<br>F(1): 5.5<br>M(1): 6.0	12.0 ± 9.3 (4)<br>F(2): 12.5 ± 10.6<br>M(2): 11.5 ± 12.0
Presenting symptoms	***(occurring in > 30% of all patients)***						
**Weakness in lower limbs (f/m, n)**	3/3 (6)	64/45 (109)	63/44 (107)	47/28 (75)	16/15 (31)	0	0/1 (1)
**Weakness in lower limbs, mean age of onset (years ±SD, n)**	9.7 ± 7.6 (3)<br>F(0)<br>M(3): 9.7 ± 7.6	16.9 ± 12.8 (103)<br>F(60): 16.4 ± 12.8<br>M(43): 17.6 ± 14.6	17.2 ± 12.8 (101)<br>F(59): 16.6 ± 12.8<br>M(42): 18.0 ± 14.5	21.2 ± 12.3 (71)<br>F(45): 19.4 ± 11.0<br>M(26): 24.3 ± 14.1	7.3 ± 7.8 (29)<br>F(14): 7.8 ± 8.6<br>M(15): 6.8 ± 7.2	0<br>F(0)<br>M(0)	20.0 (1)<br>F(0)<br>M(1): 20.0
**Proximal weakness (f/m, n)**	2/2 (4)	40/31 (71)	39/30 (69)	30/17 (47)	7/10 (17)	1/1 (2)	1/2 (3)
**Proximal weakness, mean age of onset (years ±SD, n)**	10.5 ± 10.6 (2)<br>F(0)<br>M(2): 10.5 ± 10.6	17.5 ± 12.8 (65)<br>F(35): 18.0 ± 11.6<br>M(30): 17.0 ± 14.2	18.1 ± 12.6 (63)<br>F(34): 18.4 ± 11.4<br>M(29): 17.6 ± 14.1	22.3 ± 12.2 (43)<br>F(27): 21.5 ± 10.8<br>M(16): 23.7 ± 14.5	9.3 ± 8.4 (15)<br>F(5): 7.4 ± 3.5<br>M(10): 10.3 ± 10.0	5.8 ± 0.4 (2)<br>F(1): 6.0<br>M(1): 6.0	9.3 ± 9.3 (3)<br>F(1): 5.0<br>M(2): 11.5 ± 12.0
**Hyperckaemia (f/m, n)**	4/1 (5)	30/23 (53)	29/23 (52)	17/12 (29)	9/10 (19)	1/1 (2)	2/0 (2)
**Hyperckaemia, mean age of onset (years ±SD, n)**	12.5 ± 7.8 (2)<br>F(1): 7.0<br>M(1): 18.0	10.8 ± 8.8 (50)<br>F(28): 11.3 ± 7.6<br>M(22): 10.2 ± 10.3	11.0 ± 8.8 (49)<br>F(27): 11.7 ± 7.6<br>M(22): 10.2 ± 10.3	14.6 ± 9.2 (27)<br>F(16): 15.0 ± 7.2<br>M(11): 13.9 ± 11.9	6.1 ± 5.9 (18)<br>F(8): 5.5 ± 3.5<br>M(10): 6.6 ± 7.4	5.8 ± 0.4 (2)<br>F(1):5.5<br>M(1): 6.0	12.5 ± 10.6 (2)<br>F(2): 12.5 ± 10.6<br>M(0)
Current motor function							
**Ambulant (+/- support) (f/m, n)**	129/105 (234)	128/111 (239)	123/106 (229)	99/73 (169)	23/30 (53)	1/2 (3)	3/1 (4)
**Mean age of patients able to climb stairs (+/- support) (years ±SD, n)**	36.8 ± 14.5 (78)<br>F(40): 36.0 ± 13.2<br>M(38): 37.7 ± 15.8	39.1 ± 15.0 (80)<br>F(45): 39.8 ± 17.5<br>M(35): 38.3 ± 13.8	39.3 ± 16.3 (77)<br>F(43): 40.1 ± 16.6<br>M(34): 38.2 ± 16.2	43.5 ± 14.6 (59)<br>F(37): 41.2 ± 16.1<br>M(22): 47.3 ± 10.9	25.5 ± 16.9 (15)<br>F(5): 35.1 ± 21.5<br>M(10): 20.6 ± 9.7	25.0 ± 6.0 (3)<br>F(1): 24.3<br>M(2): 25.4 ± 8.4	0<br>F(0)<br>M(0)
**Mean age of patients able to walk (+/- support) (years ±SD, n)**	38.3 ± 16.1 (115)<br>F(68): 38.0 ± 16.0<br>M(47): 38.7 ± 16.5	44.9 ± 15.4 (116)<br>F(68): 44.9 ± 14.1<br>M(48): 45.0 ± 17.3	44.9 ± 15.1 (110)<br>F(66): 44.6 ± 14.2<br>M(44): 45.3 ± 16.7	47.0 ± 13.4 (82)<br>F(50): 45.5 ± 13.1<br>M(32): 49.5 ± 13.8	37.4 ± 18.7 (25)<br>F(14): 40.3 ± 18.0<br>M(11): 33.7 ± 19.8	0<br>F(0)<br>M(0)	49.5 ± 8.0 (3)<br>F(2): 53.8 ± 4.3<br>M(1): 40.9
**Non-ambulant (f/m, n)**	43/38 (81)	47/31 (78)	44/31 (75)	21/17 (38)	21/12 (33)	0	2/2 (4)
**Wheelchair use, part-time (f/m, n)**	42/27 (69)	46/32 (78)	46/31 (77)	37/17 (54)	8/13 (21)	0	1/1 (2)
**Mean age of part-time wheelchair users (years ±SD, n)**	37.6 ± 16.5 (69)<br>F(42): 38.5 ± 16.7<br>M(27): 36.2 ± 16.4	39.9 ± 18.0 (78)<br>F(46): 41.4 ± 16.3<br>M(32): 37.9 ± 19.7	40.3 ± 17.6 (77)<br>F(46): 41.4 ± 16.3<br>M(31): 38.7 ± 19.5	45.8 ± 15.8 (54)<br>F(37): 43.6 ± 15.2<br>M(17): 50.5 ± 16.4	25.4 ± 13.6 (21)<br>F(8): 28.9 ± 16.6<br>M(13): 23.2 ± 11.6	0<br>F(0)<br>M(0)	48.9 ± 11.3 (2)<br>F(1): 56.8<br>M(1): 40.9
**Wheelchair use, full-time (f/m, n)**	49/35 (84)	52/28 (80)	49/28 (77)	23/15 (38)	24/11 (35)	0	2/2 (4)
**Mean age of full-time wheelchair users (years ±SD, n)**	40.2 ± 18.1 (84)<br>F(49): 37.2 ± 15.0<br>M(35): 44.2 ± 21.3	42.1 ± 16.4 (80)<br>F(52): 40.9 ± 16.2<br>M(28): 44.1 ± 16.8	42.5 ± 16.1 (77)<br>F(49): 41.5 ± 15.9<br>M(28): 44.1 ± 16.8	53.2 ± 12.7 (38)<br>F(23): 52.4 ± 12.9<br>M(15): 54.3 ± 12.6	32.6 ± 12.0 (35)<br>F(24): 31.5 ± 11.8<br>M(11): 34.8 ± 12.6	0<br>F(0)<br>M(0)	27.4 ± 10.2 (4)<br>F(2): 36.0 ± 3.9<br>M(2): 18.9 ± 2.2
Ventilation							
**Non-invasive (f/m, n)**	28/16 (44)	32/17 (49)	30/16 (46)	20/11 (31)	8/4 (12)	0	2/1 (3)
**Mean age of starting non-invasive ventilation (years ±SD, n)**	35.3 ± 14.0 (39)<br>F(24): 34.4 ± 12.9<br>M(15): 36.7 ± 15.9	39.4 ± 15.9 (42)<br>F(28): 39.9 ± 15.0<br>M(14): 38.4 ± 18.2	40.8 ± 15.0 (39)<br>F(26): 40.6 ± 14.9<br>M(13): 41.1 ± 15.7	46.6 ± 13.7 (24)<br>F(16): 45.5 ± 15.4<br>M(8): 48.8 ± 10.1	32.7 ± 13.2 (12)<br>F(8): 33.3 ± 11.9<br>M(4): 31.5 ± 17.3	0<br>F(0)<br>M(0)	26.6 ± 7.0 (3)<br>F(2): 30.5 ± 2.1<br>M(1): 18.8
**Invasive (f/m, n)**	3/1 (4)	7/2 (9)	7/2 (9)	3/1 (4)	4/1 (5)	0	0
**Mean age of starting invasive ventilation (years ±SD, n)**	27.5 ± 12.6 (4)<br>F(3): 23.1 ± 11.0<br>M(1): 40.8	43.1 ± 10.3 (6)<br>F(5): 43.9 ± 11.3<br>M(1): 39.0	43.1 ± 10.3 (6)<br>F(5): 43.9 ± 11.3<br>M(1): 39.0	62.0 (1)<br>F(1): 62.0<br>M(0)	39.3 ± 5.0 (5)<br>F(4): 39.4 ± 5.7<br>M(1): 39.0	0<br>F(0)<br>M(0)	0<br>F(0)<br>M(0)
